# Host-specific targets of *Histomonas meleagridis* antigens revealed by immunoprecipitation

**DOI:** 10.1038/s41598-025-88855-y

**Published:** 2025-02-17

**Authors:** Marcelo de Jesus Ramires, Karin Hummel, Tamas Hatfaludi, Michael Hess, Ivana Bilic

**Affiliations:** 1 Clinic for Poultry and Fish Medicine, Department for Farm Animals and Food System Science, University of Veterinary Medicine Vienna, Veterinärplatz 1, Vienna, A-1210 Austria; 2 VetCore Facility for Research, University of Veterinary Medicine Vienna, Veterinärplatz 1, Vienna, A-1210 Austria

**Keywords:** Histomonas meleagridis, Immunoprecipitation, LCMS, Virulence, Histomonosis, Proteomics, Parasitology, Parasite host response, Pathogens, Immunoblotting, Immunoprecipitation, Mass spectrometry, Proteomic analysis, Parasitic infection

## Abstract

*Histomonas meleagridis*, a protozoan parasite responsible for histomonosis (syn. Blackhead disease, histomoniasis), presents an increasing challenge for poultry health, particularly with the ban of licensed prophylactic and treatment options. Recent studies have explored *H. meleagridis* proteome, exoproteome, and surfaceome, linking molecular data to virulence and in vitro attenuation. Nevertheless, proteins involved in interactions with hosts remain largely unknown. In this study, we conducted immunoproteome analyses to identify key antigens involved in the humoral immune response of the parasite’s main hosts, turkeys and chickens. Immunogenic proteins were isolated via immunoprecipitation using sera from chickens and turkeys that were vaccinated with a single attenuated strain and challenged with virulent strains of the protozoan, respectively. Mass spectrometry identified 155 putative *H. meleagridis* immunogenic proteins, of which 43 were recognized by sera from both hosts. In silico antigenicity screening (VaxElan) identified 33 pan-reactive antigens, with VaxiDL further highlighting 10 as potential vaccine candidates. Comparative analysis revealed host-specific immune responses, with 16 differential immunogenic proteins in chickens (6 specific to virulent and 10 to attenuated preparations) and 19 unique proteins in turkeys, all associated with virulent strains. These results enhance our understanding of *H. meleagridis* immunogenic protein dynamics and host-pathogen specificities, supporting the development of improved diagnostic tools and potential protective measures against the infection.

**Supplementary Information:**

The online version contains supplementary material available at 10.1038/s41598-025-88855-y.

## Introduction

*Histomonas meleagridis* is an extracellular parasitic protozoan, and the etiological agent of histomonosis (syn. Blackhead disease or histomoniasis) in gallinaceous birds, characterized by inflammation and necrosis of the ceca and liver^1^. In turkeys, the infection with *H. meleagridis* often results in an acute disease that can induce high mortalities in up to 100% of the flock^2^. In chickens, the disease is less severe but still leads to substantial production losses due to a drop in egg production and increased mortality^3^. Until the beginning of this century, cases of histomonosis were well controlled by antihistomonal medication as a preventive or therapeutic intervention^4^. However, with the introduction of new drug legislation within the European Union and the USA, all options for prevention and treatment were banned due to reasons of food safety^4^. A higher prevalence of the parasite in free-range layer chickens can easily be explained by the transmission of *H. meleagridis* within eggs of the caecal nematode *Heterakis gallinarum* which is the most efficient way to spread the parasite^5^. Consequently, the prevalence of *H. meleagridis* and, by association, the occurrence of histomonosis have been increasingly reported in different husbandry systems^6^. A promising prototype live vaccine, based on a clonal attenuated in vitro culture, has been proven efficacious in preventing histomonosis^7^. However, the attenuation process seems to be strain-specific, and prospects of its commercial availability remain challenging due to constraints regarding its application^8^. As a direct consequence of the reappearance of *H. meleagridis* and the ban on all effective therapies against the infection, research on the parasite and the disease has gained new incentives^6^.

Earlier molecular studies on *H. meleagridis* focused on its phylogeny while only recently, “omics”-based investigations have taken place^8,9^. Supported by the sequences of a transcriptome database, proteome and exoproteome analyses identified differentially expressed proteins comparing a virulent and an attenuated strain of the protozoan^10–13^. A paramount contribution to molecular data for this parasite came in the form of the full genomic sequence of the two aforementioned strains^14^. Finally, a more recent addition to *H. meleagridis “*omics” research repertoire, identified its surface and surface-associated proteins^15^. These surfaceome proteins are expected to be involved in the first contact with the host, promoting the colonization of the mucosa and mechanisms for the destruction of the host’s tissues^16,17^. The identification of the molecular mechanisms by which parasites and hosts interact with each other is crucial in understanding how parasites evade the host’s immune system and ultimately cause disease. Despite all the research on *H. meleagridis*, much is still unknown about this organism regarding the genes and proteins involved in such interactions. Immunoaffinity offers a selective and efficient method for isolating protein complexes involved in the interaction with an immune system of the host and determining their composition, a knowledge, that can provide key insights into their functions^18,19^. Therefore, in the present study, we aimed to identify the key antigens involved in the antibody response of the two main hosts against *H. meleagridis*. As such, we isolated immunogenic proteins of the protozoan by immunoprecipitation employing sera from chickens and turkeys, vaccinated and infected, with an attenuated and virulent strain of *H. meleagridis*, respectively.

## Results

### Efficiency analysis of chicken and turkey immune sera

**Fig. 1:**
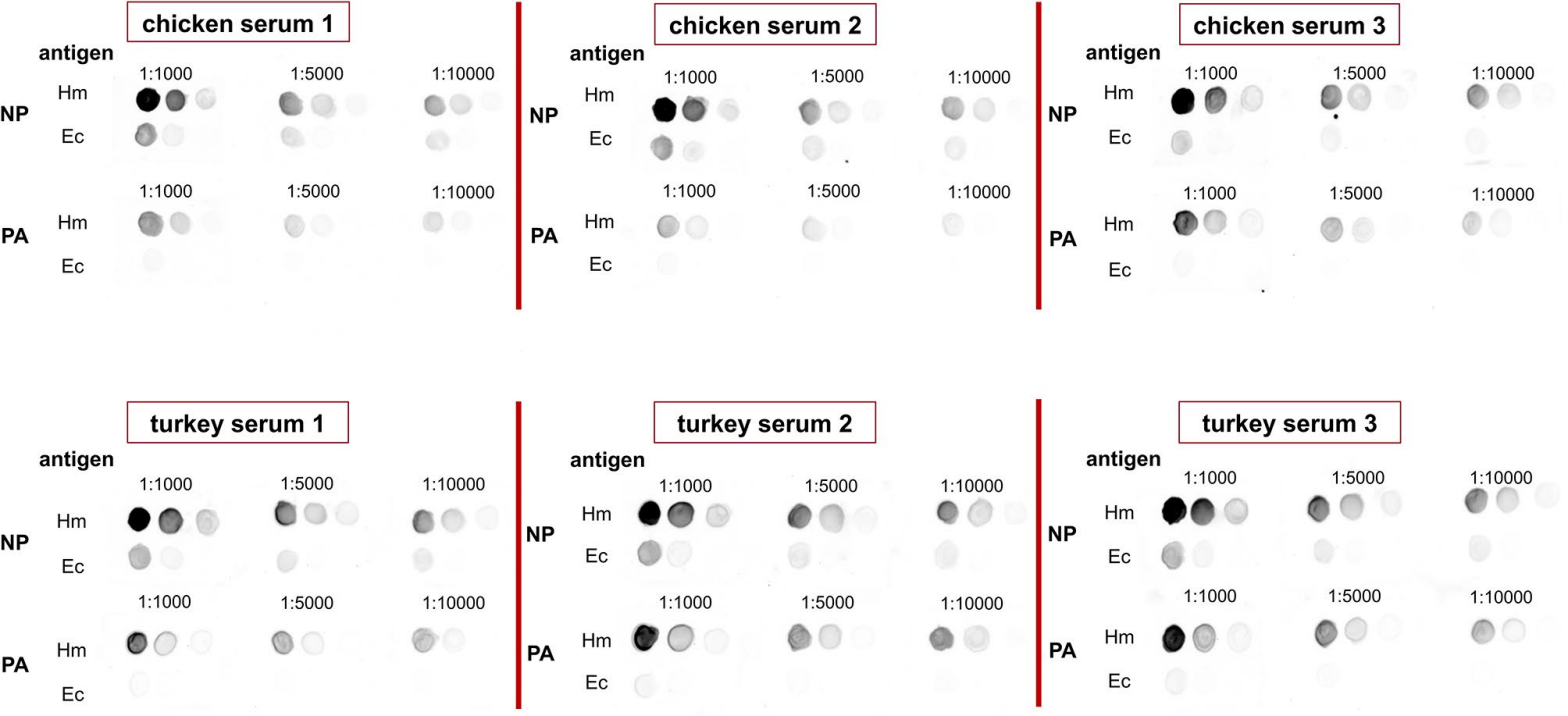
Serum reactivity testing against* H. meleagridis* and* E. coli* antigens. Dot-blot analyses to compare the reactivity of chicken and turkey sera against *H. meleagridis* (Hm) and *E. coli* (Ec) protein extracts. Sera were tested in two conditions: NP (no pre-absorption) and PA (pre-absorption with acetone powders of *E. coli* proteins to reduce background signal due to cross-reactivity with *E. coli* proteins). Dilutions at 1:1000, 1:5000, and 1:10000 are shown, for the selection of optimal serum concentrations.

To confirm the presence of anti-*H. meleagridis* antibodies and to determine the optimal dilution of each serum used for immunoproteomic assays, dot-blot analyses were performed with immune sera collected from chickens and turkeys, vaccinated and infected with *H. meleagridis*. Due to their natural presence in the gut, detection of *E. coli* proteins was expected by the sera of both bird species. Moreover, in such an experiment where *H. meleagridis* is co-cultivated with *E. coli* DH5*α*, there is a risk of a high-background signal caused by the bacterial presence, which can interfere with the accuracy of the immunoprecipitation assay. To minimize these signals, all sera were pre-absorbed with *E. coli* acetone powders, which are protein extracts prepared by precipitating *E. coli* cells with acetone and used to remove non-specific antibodies, as described in the Methods and Materials section. The results are shown in Fig. 1. To test the efficiency of this procedure, the reactivity of the pre-absorbed (PA) and non-pre-absorbed (NP) sera to various dilutions of *H. meleagridis* and *E. coli* DH5*α* proteins were carried out by dot-blot assays. Results demonstrated that the pre-absorption efficiently decreased the background signal for all tested sera, as a very low background signal was already detected after a 1:1,000 dilution and no background signal after a 1:5,000 dilution. A final dilution of 1:1,000 of all sera was used for further immunoassays, immunoprecipitation, and western blotting. Interestingly, the immuno-reaction towards the *H. meleagridis* antigen preparations was stronger in all tested turkey immune sera as compared to the tested chicken immune sera.

To ensure consistency in the experimental procedure, the same *E. coli* acetone powders were used for pre-absorption of both naïve chicken and turkey sera, from three individual birds. As no immune response was anticipated against *H. meleagridis* proteins, a dot-blot analysis was not performed. Instead, the naïve sera were used at a 1:1000 dilution in all subsequent immunoprecipitation (IP) experiments, following the established protocol for the immune sera.

### Immunoprecipitation and LCMS analyses

The immunoprecipitation was carried out using pooled sera from individual birds, either from chickens or turkeys, each vaccinated and infected with an attenuated and virulent strain of *H. meleagridis*, respectively.

**Fig. 2:**
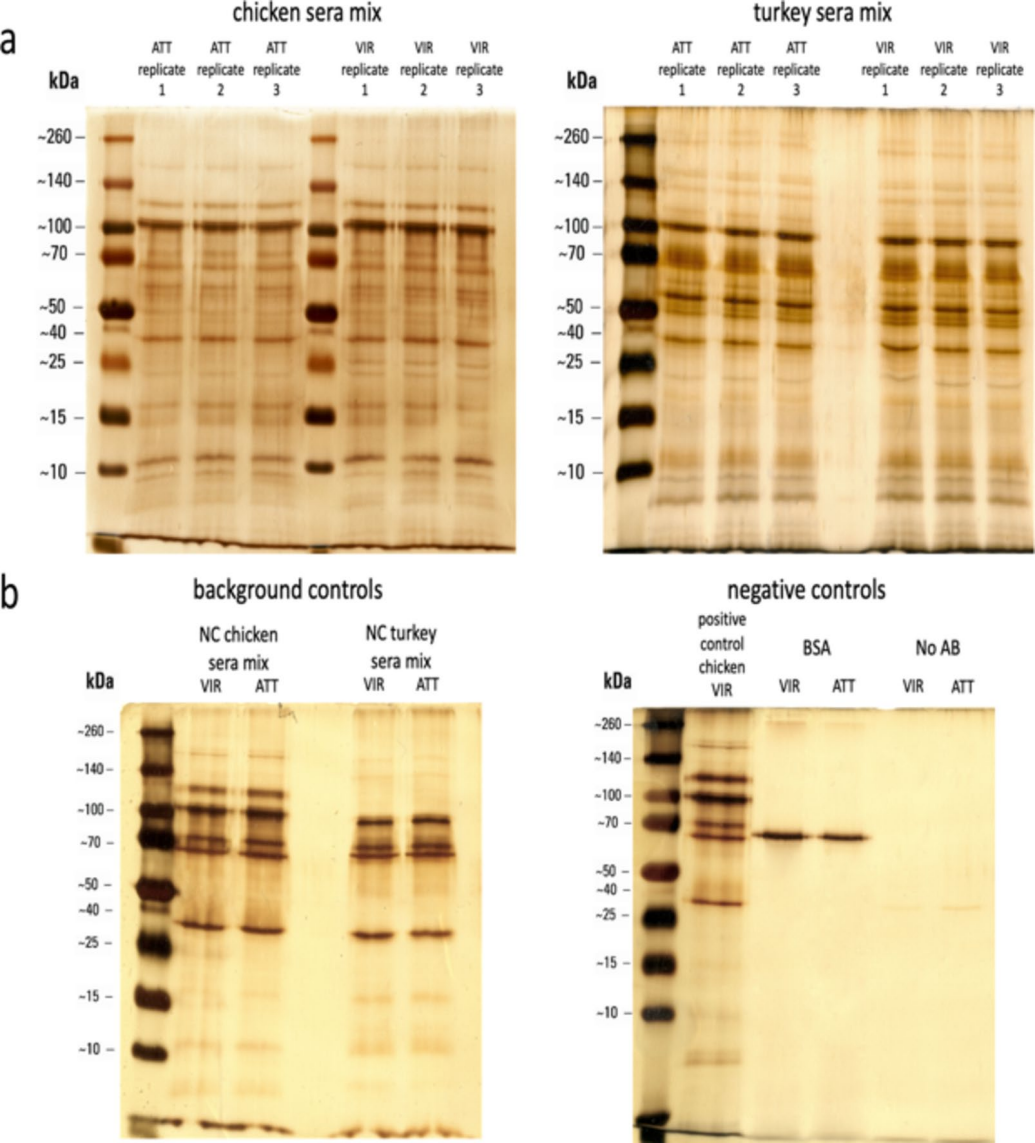
Gel electrophoresis of immunoprecipitated proteins from chicken and turkey sera and control samples. (**a**) Gel electrophoresis of proteins immunoprecipitated from chicken (left gel) and turkey (right gel) sera. Each panel shows triplicate lanes for proteins from attenuated (ATT) and virulent (VIR) *H. meleagridis* strains. The protein’s molecular weights are indicated in kilodaltons (kDa). (**b**) The left gel displays background controls from IP using sera from negative control (NC) chickens and turkeys tested with protein extracts of *H. meleagridis* from virulent (VIR) and attenuated (ATT) strains. The right gel shows negative controls, comprised of a BSA, and a no antibody (No AB) lane, in comparison to a positive control.

The SDS-PAGE analyses of immunoprecipitated (IP) samples show distinct electrophoretic profiles comparing the pooled chicken sera with the pooled turkey sera (Fig. 2a). Minor variations can be seen between the *H. meleagridis* virulent and attenuated antigenic preparations in case the immunoprecipitation was performed with the same pooled sera (Fig. 2a). To exclude proteins unspecifically cross-reacting with the immune sera, an immunoprecipitation protocol, using either the pooled sera from chickens or turkeys that were never exposed to the protozoan, was employed. The SDS-PAGE analysis following the IP assay with naïve sera demonstrated prominent unspecific binding towards specific proteins (Fig. 2b). Nevertheless, a comparison of the electrophoretic profiles of both immunoprecipitation assays shows clear distinctions between immunized and naïve sera (Fig. 2).

In addition to controls using naïve sera, two more negative controls were performed to assess the robustness of the protocol. The first negative control employed immunoprecipitation with beads coated only with BSA, whereas the second control used uncoated beads. The SDS-PAGE and LCMS analyses of immunoprecipitations using the same *H. meleagridis* antigen preparations, as in experiments with serum-coated beads, resulted in a pull-down of only BSA or an empty reaction, depending on whether BSA-coated or uncoated beads were used, respectively (Fig. 2b). This guaranteed that serum-independent coupling of antigens to the beads did not take place.

### Identification of* H. meleagridis* immunogenic proteins

**Fig. 3:**
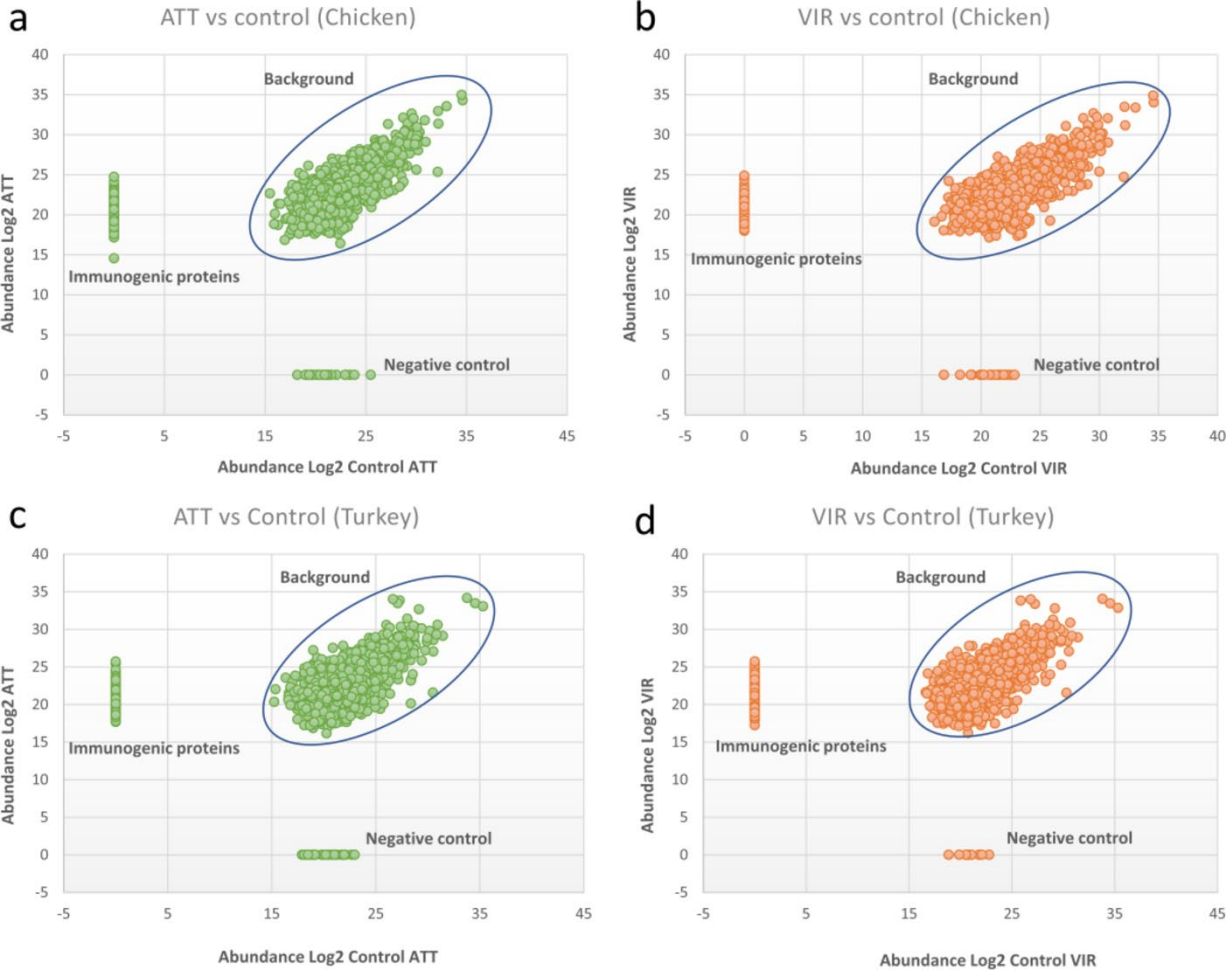
Differential protein distribution in immunoprecipitation experiments. The scatter plots depict the results from LCMS analysis of immunoprecipitation experiments, displaying the distribution of proteins across different categories: immunogenic proteins, negative control, and background. The scatter plots distinguish between proteins specific to the antibody of interest (immunogenic proteins), proteins that bind exclusively to the negative control antibody (negative control), and proteins common to both conditions (background).

**Fig. 4:**
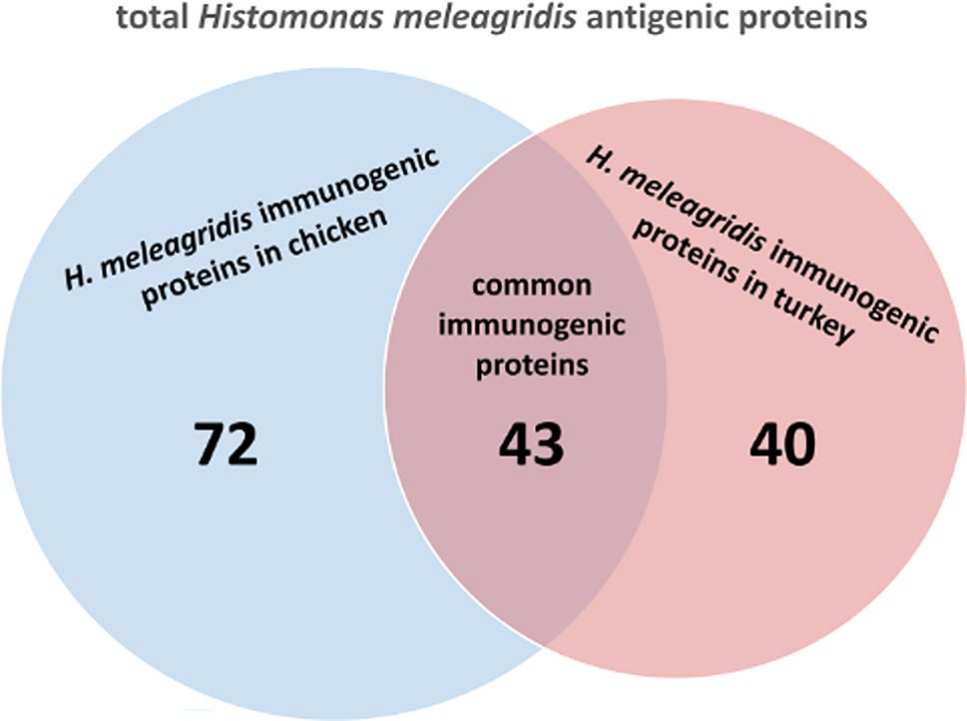
Venn diagram of immunogenic proteins in* H. meleagridis* across chicken and turkey hosts. Distribution of immunogenic proteins identified in *H. meleagridis* immunoprecipitation using sera from chickens and turkeys. The blue circle represents proteins unique to chickens (*n* = 72), the red circle represents proteins unique to turkeys (*n* = 40), and the overlapping area contains proteins common to both hosts (*n* = 43).

An initial LCMS analysis of immunoprecipitation assays identified a total of 234 proteins in experiments using the pooled chicken immune sera, and 200 proteins in experiments with the pool of turkey immune sera. LCMS analysis of immunoprecipitation assays using chicken and turkey naïve sera identified a total of 146 and 133 proteins, respectively (Fig. 3). Normalizing the results of initial LCMS analysis by excluding proteins identified in experiments with naïve sera, retained 115 and 83 proteins for further analysis, which specifically reacted with chicken and turkey anti-*H. meleagridis* immune sera, respectively (Fig. 3, Supplementary Table S1). Overall, amongst all analyzed samples, a total of 155 proteins were identified as putative *H. meleagridis* immunogenic proteins (Fig. 3, Supplementary Table S1). Out of 115 proteins identified by the chicken immune sera, 72 were not recognized by the turkey sera. Similarly, out of 83 immunogenic proteins, identified in immunoprecipitations with turkey immune sera, 40 were not recognized by chicken immune sera. A total of 43 common *H. meleagridis* immunogenic proteins were detected by both pooled sera (Fig. 4).

Overall, by combining the LCMS data from immunoprecipitation experiments using *H. meleagridis* immune and naïve sera, we were able to differentiate identified proteins into three different groups: (1) background: proteins (*n* = 119 in chicken IP, *n* = 117 in turkey IP) present in both samples, IP with sera exposed to *H. meleagridis* and IP with naïve sera (2) negative control: proteins (*n* = 36 in chicken IP, *n* = 46 in turkey IP) binding only to the naïve sera; (3) *H. meleagridis* immunogenic proteins: proteins (*n* = 115 in chicken IP; *n* = 83 in turkey IP) binding specifically to sera from birds exposed to *H. meleagridis* (Fig. 3). These categories were created based on the LCMS results, where statistical analysis was performed to identify and include only significant proteins.

**Fig. 5:**
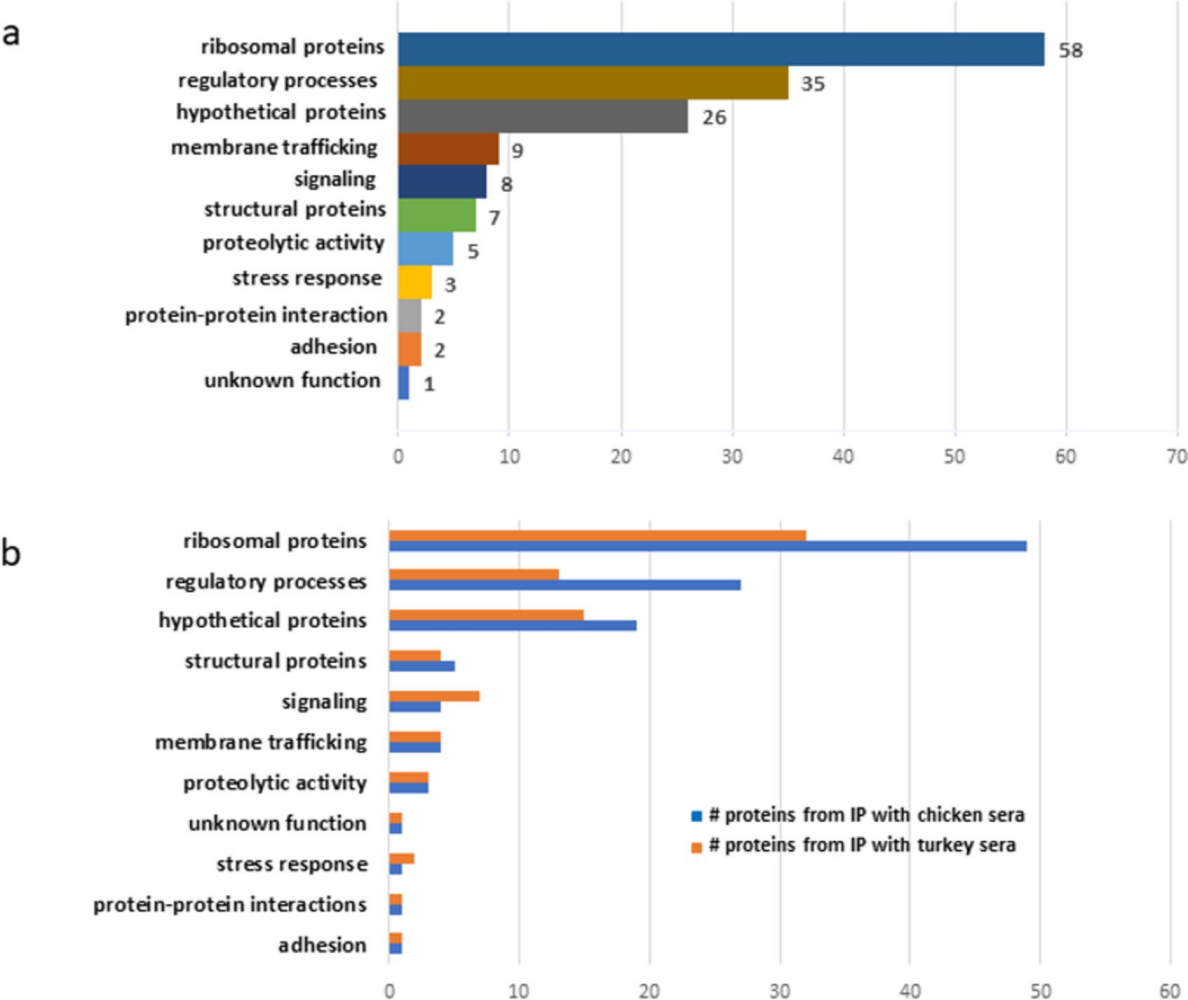
Functional annotation of* H. meleagridis* immunogenic proteins. (**a**) Functional annotation of total *H. meleagridis* immunogenic proteins assigned to categories regardless of the host. (**b**) separated into functional categories by host: chicken versus turkey.

Using BLAST analysis, we were able to sort the identified immunogenic proteins into functional groups based on their annotation (Fig. 5). The most prominent group was ribosomal proteins making up 37.4% (*n* = 58) of the total immunogenic proteins, followed by those involved in regulatory processes at 22.6% (*n* = 35), and hypothetical proteins at 16.8% (*n* = 26). Next in line were proteins associated with membrane trafficking at 5.8% (*n* = 9) and signaling proteins at 5.2% (*n* = 8). Structural proteins accounted for 4.5% (*n* = 7), proteins with proteolytic activity comprised 3.2% (*n* = 5), and stress response proteins made up 1.9% (*n* = 3) of the total. Adhesion, and proteins promoting protein-protein interactions, each group made up at 1.3% (*n* = 2). The remaining group, unknown proteins, accounted for 0.65% (*n* = 1) of the total immunogenic proteins (Fig. 5a, Supplementary Table S1).

Analyzing the data by comparing the chicken vs. turkey sera, ribosomal proteins were the most abundant immunogenic proteins recognized by sera of both species (Fig. 5b). Notable differences were observed for categories: (i) proteins involved in the regulatory processes and (ii) hypothetical proteins, in which immunogenic proteins were more frequently recognized by chicken immune sera with 17.4% (*n* = 27) and 12.3% (*n* = 19) of the total immunogenic proteins, respectively, as compared to turkey sera with 8.4% (*n* = 13) and 9.7% (*n* = 15), respectively. Conversely, proteins involved in signaling were more frequently identified as immunogenic by turkey (4.5%, *n* = 7), than by chicken immune sera (2.6%, *n* = 4) (Fig. 5b, Supplementary Table S1). The single immunogenic protein of unknown function, a DUF882 domain-containing protein (KAH0796450), was recognized by the immune sera of both hosts. Apart from these highlighted differences, the overall distribution of proteins across the other functional groups remained similar (Fig. 5b, Supplementary Table S1). A comparison with the surface proteome data^15^ showed that 71.6% (*n* = 111) of the identified immunogenic proteins have been previously described as putative surface proteins (Supplementary Table S1).

### Virulent vs. attenuated strains of* H. meleagridis*

**Fig. 6:**
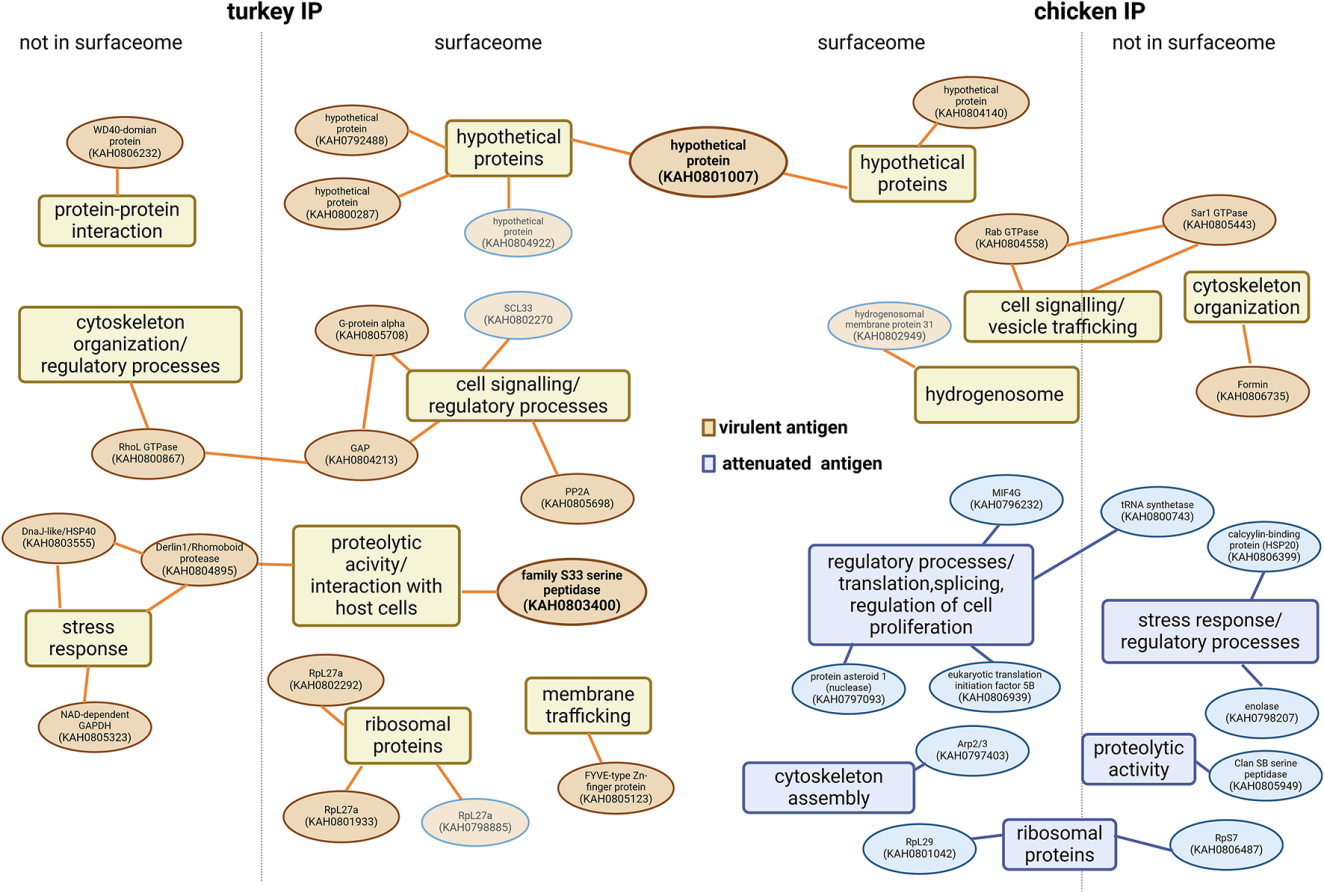
Schematic representation of* H. meleagridis* differential immunogenic proteins. Proteins detected only in the antigen preparation from the virulent strain are shown in yellow and proteins detected only in the antigen preparation from the attenuated strain in blue. The grouping of proteins was based on their functional annotation, IP experiment in which they were detected, and surface-associated position. Proteins shown in bubbles of a fading yellow color with a blue rim were also detected in the other IP experiment (chicken or turkey) but in that experiment, they were not differential as they were detected in both antigenic preparations.

Depending upon the origin of the immune serum, a substantial variation in immunogenic proteins between the virulent and attenuated strains of *H. meleagridis* was observed. When analyzed with the pool of chicken immune sera, a difference of 16 proteins was evident between the antigen preparations from the virulent and attenuated strains of *H. meleagridis* (Table 1). Out of these 16 differential immunogenic proteins, a subset of 10 proteins was uniquely present within the samples from the attenuated strain (Fig. 6; Table 1). None of these 10 proteins were detected in immunoprecipitations conducted with the pool of turkey immune sera. Conversely, the remaining 6 differential immunogenic proteins were detected in antigenic preparations from the virulent strain (Fig. 6; Table 1). Two of them, a hydrogenosomal membrane protein 31 precursor (KAH0802949) and a hypothetical protein (KAH0801007) were also identified in assays utilizing the pooled turkey immune sera (Fig. 6; Table 1; Supplementary Table S1). Interestingly, experiments using pooled immune sera from turkeys identified differential immunogenic proteins only within antigenic preparation from the virulent strain, as all immunogenic proteins discovered within the antigen preparations from the attenuated strain were concurrently present in the antigen pool of the virulent strain (Fig. 6; Table 1, Supplementary Table S1). Most of the 19 differential immunogenic proteins exclusive to the antigen preparations from the virulent strain were specifically identified by the pool of turkey immune sera (Fig. 6; Table 1, Supplementary Table S1). Only 4, two hypothetical proteins (KAH081007 and KAH0804922), a serine/arginine-rich SC35-like splicing factor SCL33 (KAH0802270), and a 60 S ribosomal protein L27a (RPL27a; KAH0798885) were also identified in immunoaffinity experiments using the pooled chicken immune sera (Fig. 6; Table 1, Supplementary Table S1). However, only one of them, a hypothetical protein (KAH0801007), was identified in both IP experiments as a differential antigen (Fig. 6; Table 1). To complement the analyses, we measured the proteins in antigenic preparations from each strain before immunoprecipitation (pre-IP antigenic preparation), identifying 1017 proteins in the attenuated strain and 1532 proteins in the virulent strain (Supplementary Table S2). Despite this, most differential antigens were not detected at significantly different levels between the strains in the pre-IP samples. Their differential abundance was only observed in the IP samples, indicating that their detection depended on the presence of specific antibodies in the pooled sera, and their selective enrichment during immunoprecipitation (Table 1).

**Table 1:** List of differential immunogenic proteins.

			Histomonas IP Chicken sera		Histomonas IP- turkey sera				antigenic preparation before IP	
Accession number	Protein name	# peptides	Attenuated antigen	Virulent antigen	Attenuated antigen	Virulent antigen	Functional annotation	Surfaceome^*^	Attenuated strain	Virulent strain
KAH0805443	Ras family GTPase Sar1	2	no	yes	no	no	signaling/vesicle trafficking	no	yes	yes
KAH0804140	hypothetical protein	2	no	yes	no	no	hypothetical protein	yes	no	no
KAH0804558	Ras family protein GTPase Rab	2	no	yes	no	no	signaling/vesicle trafficking	yes	no	yes
KAH0806735	Formin-like 2 Domain containing protein	2	no	yes	no	no	regulatory processes	no	no	no
KAH0802949	hydrogenosomal membrane protein 31, precursor	2^§^; 4^$^	no	yes	yes	yes	regulatory processes/hydrogenosome	yes	no	yes
KAH0801007	hypothetical protein	2^§^; 3^$^	no	yes	no	yes	hypothetical protein	yes	no	no
KAH0796232	MIF4G domain-containing protein	2	yes	no	no	no	regulatory processes	yes	yes	yes
KAH0797093	protein asteroid 1	7	yes	no	no	no	regulatory processes	yes	yes	yes
KAH0806939	eukaryotic translation initiation factor 5B	4	yes	no	no	no	regulatory processes	yes	yes	yes
KAH0805949	clan SB, family S8, subtilisin-like serine peptidase	5	yes	no	no	no	proteolytic activity	no	yes	no
KAH0801042	ribosomal protein L29	5	yes	no	no	no	ribosomal protein	yes	no	no
KAH0806487	40 S ribosomal protein S7	3	yes	no	no	no	ribosomal protein	no	no	no
KAH0800743	tRNA binding domain containing protein	3	yes	no	no	no	regulatory processes	no	yes	yes
KAH0806399	calcyclin-binding protein-like (HSP20)	2	yes	no	no	no	stress response/regulatory processes	no	yes	yes
KAH0798207	enolase family protein	2	yes	no	no	no	stress response/regulatory processes	no	yes	yes
KAH0797403	actin-related protein 2/3 complex, subunit 1	2	yes	no	no	no	structural/cytoskeleton	yes	no	yes
KAH0805698	serine/threonine-protein phosphatase alpha-2 isoform (PP2A)	2	no	no	no	yes	regulatory processes	yes	yes	yes
KAH0805708	guanine nucleotide-binding protein subunit alpha	2	no	no	no	yes	signaling	yes	yes	yes
KAH0804922	hypothetical protein	^§ ^5; 4^$^	yes	yes	no	yes	hypothetical protein	yes	no	no
KAH0805123	FYVE-type zinc finger-containing protein	2	no	no	no	yes	membrane trafficking transport	yes	yes	yes
KAH0805323	NAD-dependent glycerol-3-phosphate dehydrogenase family protein	2	no	no	no	yes	stress response/regulatory processes	no	yes	yes
KAH0804895	Derlin-1	2	no	no	no	yes	stress response/proteolytic activity	no	no	no
KAH0804213	GTPase-activator protein	2	no	no	no	yes	signalling/regulatory processes	yes	no	no
KAH0803555	dnaJ-like subfamily B member 1-like	6	no	no	no	yes	stress response	no	no	no
KAH0792488	hypothetical protein	2	no	no	no	yes	hypothetical protein	no	no	no
KAH0803400	Clan SC, family S33, methylesterase-like serine peptidase	2	no	no	no	yes	proteolytic activity	yes (up-regulated in virulent strain)	yes	yes
KAH0802270	serine/arginine-rich SC35-like splicing factor SCL33	2; 3^$^	yes	yes	no	yes	regulatory processes	yes	no	no
KAH0802292	60 S ribosomal protein L27a	8	no	no	no	yes	ribosomal protein	yes	no	no
KAH0801933	60 S ribosomal protein L27a	9	no	no	no	yes	ribosomal protein	yes	no	no
KAH0800867	ras-like GTP-binding protein RhoL isoform X2	2	no	no	no	yes	regulatory processes	no	no	yes
KAH0800287	hypothetical protein	2	no	no	no	yes	hypothetical protein	yes	no	no
KAH0798885	60 S ribosomal protein L27a	8^§^; 10^$^	yes	yes	no	yes	ribosomal protein	yes	no	yes
KAH0806232	WD repeat-containing protein 19	2	no	no	no	yes	protein-protein interactions	no	no	no

^*^Ramires et al. 2022.

^§^Chicken IP dataset.

^$^Turkey IP dataset.

### In silico analysis of antigenicity and prediction of vaccine candidates

To validate the antigenic potential of proteins identified by immunoprecipitation, bioinformatic analysis using VaxElan software analysis was performed. Further classification of the identified proteins for their potential to serve as putative vaccine candidates was carried out in the analysis using Vaxi-DL software. Since the majority of *H. meleagridis* surface proteins lack the conventional characteristics common to surface-associated proteins^15^, these predictions were excluded from consideration in the VaxElan search. Instead, the results were compared with the available surfaceome database^15^ (Supplementary Table S1).

VaxElan prediction analysis for the 115 *H. meleagridis* immunogenic proteins identified with chicken immune sera classified 74.8% (*n* = 86) as putative antigenic proteins. Additionally, VaxiDL software predicted that 24.3% (*n* = 28) could be used as possible vaccine candidates. However, since not all 28 immunogenic proteins were identified in the surface proteome database, a prerequisite for a vaccine candidate, only 18.3% (*n* = 21) could be classified as *H. meleagridis* putative vaccine candidates in chickens (Supplementary Table S1).

In a similar analysis performed on 83 *H. meleagridis* immunogenic proteins identified using turkey immune sera, VaxElan analysis predicted 78.3% (*n* = 65) of proteins to demonstrate potential antigenicity. Additionally, the VaxiDL software prediction showed that 25.3% (*n* = 21) could serve as potential vaccine candidates. Following a comparison with the *H. meleagridis* surface proteome database, we concluded that 18.1% (*n* = 15) were potential *H. meleagridis* vaccine candidates in turkeys (Supplementary Table S1). Finally, by comparing in silico antigenicity for *H. meleagridis* immunogenic proteins, a total of 33 proteins with VaxElan scores ≥ 0.5 were identified as promising pan-reactive antigens, as they were recognized by immune sera from both hosts (Table 2). Analysis with VaxiDL software narrowed down this selection to 10 proteins, identified as the most promising vaccine candidates (Table 2).

**Table 2:** List of* H. meleagridis* pan-reactive antigens and potential vaccine candidates.

accession number	Protein name	Functional annotation	VaxElan prediction	Vaxi-DL prediction	Probability (Vaxi-DL)	Surfaceome^*^
KAH0796226	40 S ribosomal protein S17-B	ribosomal protein	0.33	not a vaccine candidate	99.52	yes
KAH0806327	60 S ribosomal protein L6	ribosomal protein	0.5	not a vaccine candidate	99.61	no
KAH0796450	DUF882 domain-containing protein	unknown function	0.5	vaccine candidate	73.59	yes
KAH0796603	hypothetical protein	hypothetical protein	0.5	not a vaccine candidate	61.33	no
KAH0796674	Clan SC, family S33, methylesterase-like serine peptidase	proteolytic activity	0.5	not a vaccine candidate	99.67	yes
KAH0796712	V-type proton ATPase subunit d 2	membrane trafficking transport	0.5	not a vaccine candidate	99.39	yes
KAH0796745	40 S ribosomal protein S14	ribosomal protein	0.5	not a vaccine candidate	99.66	yes
KAH0796766	ribosomal protein L38e	ribosomal protein	0.33	not a vaccine candidate	60.1	no
KAH0806053	60 S ribosomal protein L24	ribosomal protein	0.5	not a vaccine candidate	99.3	yes
KAH0796864	ras-related C3 botulinum toxin substrate 2	signaling	0.5	vaccine candidate	98.68	yes
KAH0796912	60 S ribosomal protein L26-1	ribosomal protein	0.33	not a vaccine candidate	99.88	no
KAH0796962	ribosomal protein L31e	ribosomal protein	0.5	not a vaccine candidate	96.22	no
KAH0805986	ribosomal protein L27e	ribosomal protein	0.5	vaccine candidate	97.62	yes
KAH0805851	40 S ribosomal protein S26	ribosomal protein	0.33	vaccine candidate	76.87	yes
KAH0805668	hypothetical protein	hypothetical protein	0.5	not a vaccine candidate	97.27	yes
KAH0804850	ribosomal protein S19e	ribosomal protein	0.33	not a vaccine candidate	99.8	yes
KAH0803697	40 S ribosomal protein S18	ribosomal protein	0.33	not a vaccine candidate	98.13	yes
KAH0803326	phosphofructokinase family protein	regulatory processes	0.5	not a vaccine candidate	99.56	yes
KAH0803233	60 S ribosomal protein L33-A	ribosomal protein	0.5	not a vaccine candidate	84.61	no
KAH0802750	hypothetical protein	hypothetical protein	0.5	not a vaccine candidate	99.74	yes
KAH0802062	hypothetical protein	hypothetical protein	0.5	vaccine candidate	99.64	yes
KAH0801972	40 S ribosomal protein S2-1	ribosomal protein	0.5	not a vaccine candidate	60.74	no
KAH0801868	40 S ribosomal protein S1	ribosomal protein	0.5	vaccine candidate	72.04	no
KAH0801934	Rpl16ap	ribosomal protein	0.5	not a vaccine candidate	98.99	yes
KAH0801608	60 S ribosomal protein L9	ribosomal protein	0.5	not a vaccine candidate	99.65	yes
KAH0801475	hypothetical protein	hypothetical protein	0.5	not a vaccine candidate	99.79	yes
KAH0806883	Rab GTPase	regulatory processes	0.67	vaccine candidate	98.56	yes
KAH0800690	hypothetical protein	hypothetical protein	0.5	vaccine candidate	99.27	yes
KAH0800691	Rab GTPase	regulatory processes	0.5	vaccine candidate	86.1	yes
KAH0800779	tubulin alpha chain	structural	0.5	not a vaccine candidate	97.84	no
KAH0800347	ribosomal protein L29	ribosomal protein	0.33	not a vaccine candidate	90.71	yes
KAH0799721	Rpl16ap	ribosomal protein	0.5	not a vaccine candidate	99.3	no
KAH0799138	ABC transporter E family member 2	membrane trafficking transport	0.33	not a vaccine candidate	99.77	yes
KAH0799167	ribosomal protein	ribosomal protein	0.5	not a vaccine candidate	95.47	yes
KAH0798645	60 S ribosomal protein L7-2	ribosomal protein	0.5	not a vaccine candidate	98.49	yes
KAH0798135	40 S ribosomal protein S2-1	ribosomal protein	0.33	not a vaccine candidate	87.21	yes
KAH0798070	40 S ribosomal protein S3a	ribosomal protein	0.5	vaccine candidate	92.57	yes
KAH0797406	6-phosphofructokinase	regulatory processes	0.5	not a vaccine candidate	99.72	yes
KAH0804922^$^	hypothetical protein	hypothetical protein	0.5	not a vaccine candidate	91.79	yes
KAH0802949^§^	hydrogenosomal membrane protein 31 precursor	regulatory processes	0.5	not a vaccine candidate	99.75	yes
KAH0802270^$^	serine/arginine-rich SC35-like splicing factor SCL33	regulatory processes	0.5	vaccine candidate	95.87	yes
KAH0801007^§$^	hypothetical protein	hypothetical protein	0.67	vaccine candidate	99.16	yes
KAH0798885^$^	60 S ribosomal protein L27a	ribosomal protein	0.33	not a vaccine candidate	95.59	yes

A VaxELAN score of ≥ 0.5 suggests that the protein is antigenic. The Vaxi-DL software search to determine potential vaccine candidates used an organism model: protozoa.

^*^According to Ramires et al.2022.

^§^Differential antigen in IP with chicken sera.

^$^Differential antigen in IP with turkey sera.

## Discussion

Infections caused by *H. meleagridis* exhibit different patterns of progression in chickens and turkeys, which can be attributed to variations in the immune response mounted by these two species against the parasite^20–22^. It has been established that a prompt local immune response, which prevents the systemic dissemination of the parasite, is crucial in preventing histomonosis^23^. Cell-mediated immunity has been found to play a significant role in both chickens and turkeys, rather than the production of parasite-specific antibodies in the serum^20–22^. However, the extent to which mucosal antibodies contribute to local immunity in the intestinal tissue remains unclear. While studies have reported an increase in *H. meleagridis*-specific antibodies in parasitized intestinal tissue in chickens, comparable data for turkeys are lacking^24^. Moreover, there is limited knowledge regarding parasite proteins that trigger the immune response in the host. Therefore, the objective of this study was to employ an immunoaffinity assay coupled with mass spectrometric identification to identify and characterize these proteins.

A previous study on *H. meleagridi*s-specific immunogenic proteins showed that immune sera from chickens and turkeys show variation among individuals with respect to recognizing different parasite antigens^25^. Therefore, to comprehensively analyze the *H. meleagridis* immunoproteome, the present study utilized a pool of immune sera from three individual birds in all immunoaffinity experiments. Negative controls, including uncoated beads, beads coated with BSA only, and beads coated with chicken or turkey sera naïve for *H. meleagridis*, were also employed to ensure the robustness of the immunoaffinity system. The first two negative controls confirmed that in the implemented system, binding of the protein to the beads occurred only via antibodies. However, negative controls, using naïve sera for coating the beads, resulted in the unspecific pull-down of multiple *H. meleagridis* proteins. This phenomenon was not unexpected, as previous studies have reported high background signals in immunological assays in chickens using sera from naïve birds, indicating their cross-reaction with numerous proteins^26–28^. The exact explanation for this phenomenon is not fully understood, but it is suggested that a serum fraction containing different protein profiles, unrelated to IgG, may be responsible for the effect^28^.

In this study, the identification of *H. meleagridis*-specific antigens was based on the comparison of proteins from infected animals that cross-react with sera from birds naïve to *H. meleagridis*. Our findings show a clear distinction in profiles of immunogenic proteins depending on whether the employed pooled sera originated from chickens or turkeys. This is not surprising considering the characteristic progression of an infection with *H. meleagridis* in each host species, revealed by clinical signs, macroscopic lesions, and ultimately, immune reactions, which are altogether more severe in turkeys^23^. Even though chicken sera displayed weaker signals in dot-blot tests than turkey sera, which indicated lower antibody titer, they immunoreacted with more antigens in total. This was reflected in the higher number of immunogenic proteins detected by the chicken immune sera only. As all the immune sera used in this study originated from birds with similar exposure to the antigen (vaccination with an attenuated strain followed by challenge with a virulent parasite strain), the observed differences in the immune response seem to be due to variations within the host’s immune system. Chickens having prompter immune reactions than turkeys also seem to react against a broader antigenic spectrum. The results presented here also show that in addition to a general set of *H. meleagridis* antigens, each host species immuno-reacts against a set of specific antigens.

The absence of several immunoprecipitated proteins in the pre-IP proteome dataset generated from the same protein extracts is an intriguing finding. This suggests that some proteins, although present, were not detected in the pre-IP samples, likely due to their low concentrations being masked by more abundant proteins, highlighting limitations in the detection capabilities of current proteomic techniques. Low-abundance proteins may go undetected without additional enrichment, as a single proteomic analysis typically provides a snapshot rather than a dynamic overview of the proteome. This discrepancy could also reflect biological factors such as transient protein expression or post-translational modifications that hinder their detection in standard proteomic analyses.

Furthermore, a comparative analysis of immunogenic proteins between a virulent and an attenuated antigenic preparation demonstrated variations depending on the host. Most differential immunogenic proteins were only detected with either chicken or turkey sera, underlying a specific immune reaction of each host. Only five antigens were detected with immune sera of both bird species. Among these, a hypothetical protein (KAH0801007), demonstrated strain-specific antigenicity in both IP experiments, as it was detected only within the virulent antigenic preparation. Notably, this protein was not detected in pre-IP antigenic preparations from either strain via LCMS, suggesting its presence in very low amounts. Conversely, it was identified in the surfaceome of both strains^15^. These results imply that certain enrichment, either via immunoaffinity or selection for surface proteins was a prerequisite for its detection in LCMS measurement. Since neither surfaceome nor LCMS of pre-IP preparations indicate differential expression, it seems that observed differential antigenicity might stem from strain-specific post-translational modification (PTM), which often positively influences the immunogenicity of an antigen.

Using chicken sera, six immunogenic proteins from the virulent and ten from the attenuated strain were identified as differentially immunogenic, whereas the immunoaffinity assay using turkey sera identified strain-specific immunogens only within the virulent antigen preparation. Several factors potentially contributed to such a striking outcome. The identified strain-specific immunogenic proteins could be a result of differential gene expression or strain-specific PTMs. Available gene expression data for the same virulent and attenuated *H. meleagridis* strains indicate differential expression for only one antigen, a Clan SC, family S33, methylesterase-like serine peptidase (KAH0803400)^11,15^. This S33 serine peptidase was found up-regulated in the proteome and surfaceome of the virulent strain^15^, which corroborates the findings of the present investigation. However, the majority of identified differential immunogenic proteins do not seem to be regulated in these two strains. The parasite’s true gene expression during the invasion of the host is an unknown parameter that likely differs from the in vitro conditions measured in omics studies. Considering an entirely different course of *H. meleagridis* infection between turkeys and chickens, it is conceivable that the parasite’s gene expression varies accordingly. Variations in gene expression in vivo could affect the availability of certain antigens for the immune reaction of the host. This might account for the striking difference in the palette of immunogens that are recognized by immune sera from chickens and turkeys. In addition to the host species’ influence on the parasite’s gene expression, the local microbiome should be considered too. The described interplay between *H. meleagridis* and surrounding bacteria and microbiome dysbiosis in infected birds supports this view^9,29^. In this context, the feed could have had an influence too, as it was shown to impact on horizontal transmission of the parasite albeit variations between experiments argue for additional studies^30^. Finally, a closer examination of differential immunogenic proteins could provide insight into the molecular mechanisms that parasite uses to invade and inhabit the host. Especially in the case of the turkey IP experiment, in which differential immunogens were only detected within the virulent antigen preparation, identified proteins which might represent true virulence factors. As mentioned above, the Clan SC, family S33, methylesterase-like serine peptidase, identified as a differential antigen in the turkey IP experiment, was upregulated in the virulent strain of *H. meleagridis* in both proteome and secretome studies^11,15^, suggesting a unique role in virulence. In protozoan parasites such as *Trichomonas vaginalis* or *Entamoeba histolytica*, peptidases, particularly cysteine and serine peptidases, are implicated in a plethora of pathological processes, including the degradation of host protective barriers and the facilitation of tissue invasion^31–33^. The identification of the FYVE-type zinc finger-containing protein in the virulent sample of turkey IP, along with Rab GTPase, and Sar1 GTPase in chicken IP experiment, is of noteworthy interest. These proteins, commonly associated with vesicular trafficking, underscore the importance of endocytosis, a process crucial for successful host cell invasion and nutrient acquisition, as evidenced in parasites like *E. histolytica*^34^. The detection of signaling proteins, including the G-protein alpha subunit, GTPase-activator protein (GAP), and RhoL GTPase, in the virulent sample warrants further investigation. While their predicted functions do not suggest direct molecular interactions, the potential overlap in their roles in various cellular processes cannot be dismissed. In protozoan parasites, small GTPases are crucial for a range of cellular functions, particularly those related to tissue invasion and survival within the host, which are vital for the organism’s pathogenicity^35–37^. Their presence in the virulent strain implies possible modulation of signaling pathways that enhance the parasite’s capacity to invade, adapt, and proliferate within the host environment.

The last factor potentially influencing the results of differential immunogens detected by turkey sera, is that in contrast to chickens, turkeys received a heterologous *H. meleagridis* as a challenge strain. This strain was propagated in vitro as a xenic culture, a factor shown to increase the virulence, which in turn would stimulate a higher antibody titer^27,38^. Indeed, the stronger reaction of turkey as compared to chicken immune sera in the dot-blot screening corroborates such observations. Both *H. meleagridis* strains used in the turkey animal experiment, attenuated and virulent, are classified as the same genotype showing 0.6% divergence based on the 18 S rRNA^39,40^. Given this high degree of similarity, we believe that these genetic differences are unlikely to significantly impact the experimental outcome. Furthermore, the virulent antigen preparation used in all immunoprecipitation experiments was isogenic to the attenuated strain.

Aside from differential immunogenic proteins, this study defines a subset of 43 proteins identified in all immunoaffinity experiments using *H. melegaridis* immune sera, regardless of the host species. Out of these, 33 proteins were classified as potentially suitable vaccine candidates using VaxElan software analysis. Considering that the approach applied in the present study does not disturb macromolecular complexes, the identification of proteins that do not directly bind to the antibody was expected. While serum antibodies are not the primary factor in the development of protective immunity against *H. meleagridis*, they are useful for detecting infection in poultry, particularly in chickens that may not exhibit noticeable clinical symptoms or severe post-mortem lesions^27^. Current serodiagnostic tests for identifying *H. meleagridis* positive flocks are complex and require specific strains and antibodies that are in limited supply and not commercially available, complicating their application in different laboratory settings^27,41^.

To date, only one ELISA assay based upon recombinant proteins to detect *H. meleagridis* infections has been reported^42^. The immunogenic proteins identified in this study could help design more sensitive and specific tests with reduced cross-reactivity. However, further research is needed to confirm the immunogenicity of these proteins for sero-diagnostics. Among the pool of good antigens, VaxiDL software identified 10 potential vaccine candidates, offering valuable insights for developing subunit vaccines, especially since no commercial vaccine is currently available for *H. meleagridis*. It’s important to note that both VaxElan and VaxiDL pipelines were primarily trained on human data. While these tools can provide valuable insights, their use in a poultry pathogen context still requires further species-specific training and validation. Nevertheless, we believe that their ability to identify antigens of interest in *H. meleagridis* offers a good starting point for subsequent studies.

Another important consideration in the context of host–parasite interactions is that antigens exposed to humoral immune selection frequently undergo a dynamic antigenic variation^43^. This adaptive strategy, described in protozoan pathogens such as *T. vaginalis*^44^ and *Giardia lamblia*^45^, involves alterations in surface proteins that help these parasites to evade recognition by host antibodies. It remains plausible that *H. meleagridis* could employ similar mechanisms to evade the host immune response, hence investigations focusing on the patterns of antigen expression, as well as potential genetic or epigenetic shifts in antigenic profiles, could offer valuable insights.

In conclusion, this study elucidates the proteins of *H. meleagridis* involved in interactions with the host humoral immune system, highlighting unique immunogenic proteins that vary between the two primary hosts, chickens and turkeys. The identification of proteins specifically detected in the virulent antigenic preparation offers insights into the molecular mechanisms utilized by the parasite for host invasion and colonization, underscoring their role as true virulence factors.

Moreover, the identification of strain-specific antigens provides an important steppingstone toward understanding the molecular virulence of *H. meleagridis* and can offer practical pathways for vaccine development. In particular, proteins detected exclusively in the virulent strain may present key structural features or post-translational modifications required for efficient host colonization and parasite dissemination, making them interesting targets for vaccine development. Because histomonosis involves a complex interplay of humoral and cell-mediated immune responses, subunit vaccines targeting these virulence-associated proteins could be developed to enhance protective T-cell responses^23^. This strategy might be an improvement on the currently attenuated vaccine approaches under development, especially if the candidate proteins are capable of eliciting robust local and systemic immune responses that can prevent the parasite’s dissemination. Nevertheless, future in vivo validation of these strain-specific antigens will be essential in assessing their potential as effective vaccine candidates.

Future research should also focus on investigating the immunogenic characteristics of the genetically different *H. meleagridis* strains and comparing them with the current dataset. This comparative analysis can provide deeper insights into the immune responses elicited by the different genotypes and support the development of more effective, control strategies to combat this infection.

Our findings not only enhance the current understanding of histomonal antigenic protein dynamics but can also facilitate the development of new and improved diagnostic methods, and tailored subunit vaccines, which could significantly advance the control of this infection in poultry.

## Materials and methods

### Strains and in vitro cultivation of* H. meleagridis*

All experiments within this work were carried out using, clonal virulent and attenuated strains of *Histomonas meleagridis*, which originated from the same lineage of the protozoan, namely *H. meleagridis*/Turkey/Austria/2922-C6/04-10x/18x-DH5*α* and *H. meleagridis*/Turkey/Austria/2922-C6/04-290x/52x-DH5*α*, respectively^38^. Both strains were propagated in vitro as monoxenic mono-eukaryotic cultures together with the bacterial strain *E. coli* DH5*α*. The cultures were incubated at 40 °C in 28 mL of RPMI Media 1640 (Gibco™, Invitrogen, Vienna, Austria) with 15% heat-inactivated fetal bovine serum (FBS) (Gibco™, Invitrogen, Vienna, Austria) and 0.25% of sterilized rice starch (Carl Roth GmbH + Co. KG, Karlsruhe, Germany) and passaged every 48 h.

### Preparation of* H. meleagridis* antigen

Before harvesting, the parasites were cultivated in T-75 flasks (Sarstedt, Vienna, Austria) at 40 °C for 48 h. Upon reaching the collection time point, the cells were transferred into 50 mL falcon tubes (Sarstedt, Vienna, Austria) and centrifuged at 200×g for 5 min at room temperature. Upon completion, the supernatant containing most of the bacteria was discarded. To ensure the further removal of *E. coli* DH5*α* from the culture, the pelleted histomonads were washed four times with 45 mL of pre-warmed RPMI medium and centrifuged with the same conditions as above.

**Fig. 7:**
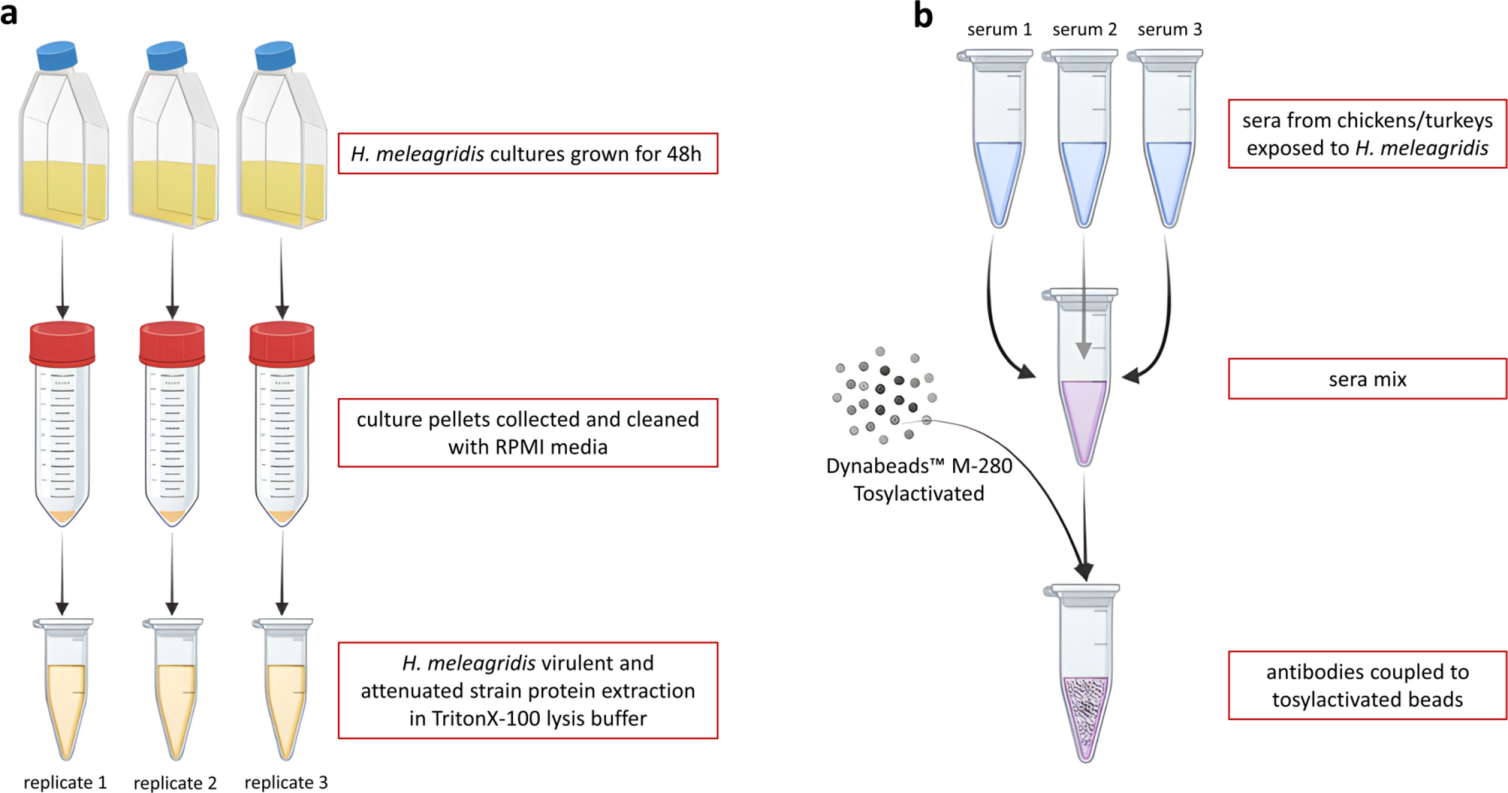
Schematic representation* H. meleagridis* cultivation and preparation of antibody-coupled beads. (**a**) Cultivation and processing: *H. meleagridis* cultures were grown for 48 h, followed by collection and cleaning of the culture pellets using RPMI medium. The proteins from virulent and attenuated strains were then extracted using Triton-X100 lysis buffer, across three replicates for each strain. (**b**) Antibody coupling: Three serum samples from chickens and turkeys, both exposed and unexposed to *H. meleagridis*, are combined into separate pools, by exposure status and host type. Antibodies from the sera were coupled to Dynabeads™ M-280 Tosylactivated beads for subsequent analysis.

Subsequently, the final pellet was resuspended in 1 mL of Triton X-100 lysis buffer (50mM Tris/HCl (pH7.4), 150mM NaCl, 1mM EDTA, 1% Triton X-100) (Merck GmbH, Vienna, Austria) with Complete protease inhibitors (Roche, Vienna, Austria), and aliquoted into two 2 mL Eppendorf tubes (Sarstedt, Vienna, Austria). To ensure cell lysis, the samples were homogenized twice at 28 Hz for 2 min using TissueLyser II (Qiagen, Hilden, Germany). The cell lysate was centrifuged at 10,000×g for 2 min at + 4 °C. The supernatant containing the *H. meleagridis* proteins was collected and used as an antigen mixture for the immunoprecipitation experiments. Reproducibility of the protocol was ensured with three technical and biological replicates prepared for each strain, grown separately in T75 culture flasks (Fig. 7a).

### Chicken and turkey anti-* H. meleagridis* immune sera

In the present study anti-*H. meleagridis* immune sera collected from animal trials^46^ (Hatfaludi et al. manuscript in preparation) were used. The selection of sera was based on available ELISA data. Furthermore to confirm the presence of anti-*H. meleagridis* antibodies the selected sera was re-tested by dot-blots before being applied in immunoprecipitation experiments. To ensure the survival of birds for the collection of sufficient antisera, it was crucial to address the severe nature of *H. meleagridis* infections in turkeys, which typically result in high mortality within three weeks post-infection. Therefore, turkeys were vaccinated with an attenuated strain of the pathogen before being challenged with the virulent strain. A similar protocol was applied to both chickens and turkeys. In detail, for turkey sera, commercial turkeys (Hybrid Converter, Miko GmbH, Austria) were vaccinated on the 1st day of life using the attenuated strain of *H. meleagridis* (*H. meleagridis*/Turkey/Austria/2922-C6/04-290x/5x-DH5*α*/55x-4CEF), that was passaged in vitro for 350 passages (350x)^47^. At 12 weeks of life, birds were infected with the virulent strain of *H. meleagridis*/Turkey/Germany/4114-C16/05, that was grown as xenic culture and passaged in vitro for 5 passages (5x) (Hatfaludi et al., manuscript in preparation). At 15 weeks of life, which was 3 weeks post-infection, immune sera were collected. For chicken sera, specific pathogen-free (SPF) chickens (VALO, Lohmann, Cuxhaven, Germany) were vaccinated on the 1st day of life using the attenuated strain *H. meleagridis*/Turkey/Austria/2922-C6/04-290x/13x-DH5*α* that was passaged in vitro for 303 passages (303x). Five weeks later, birds were infected with the virulent strain *H. meleagridis*/Turkey/Austria/2922-C6/04-10x/18x-DH5*α*, passaged in vitro for only 28 passages (28x). At 5 weeks post-infection, the immune sera from vaccinated and infected chickens were collected.

The animal trials and all the included procedures on experimental birds were discussed and approved by the institutional ethics committee and licensed by the Austrian government. For turkeys, under the license number: GZ 2020 − 0.028.651. For chickens, under the license numbers 68.205/0161-WF/V/3b/2017 and GZ 2020–0.220.316. All methods were performed in accordance with the relevant guidelines and regulations. Additionally, this study is reported in accordance with ARRIVE guidelines (https://arriveguidelines.org).

### Chicken and turkey naïve sera

Sera from 3 uninfected SPF chickens, collected at 5 weeks of age, were used as a negative control in all experiments^48^. Similarly, sera from 3 uninfected commercial turkeys, were collected at 12 weeks of age (Hatfaludi et al., manuscript in preparation). The absence of histomonads in turkeys was proven by continuous cloacal swabbing and PCR analysis. SPF chickens remained isolated for the duration of the animal trial, with no possibility of contact with the protozoa.

### Pre-absorption of serum samples against* E. coli* DH5 α

To decrease *E. coli* background reactions, pre-absorption of the sera was performed^25^. For this purpose, serum samples were incubated with acetone powders made from *E. coli* DH5*α*, the bacteria cohabitating with *H. meleagridis* in the mono-eukaryotic culture. For that, 100 µl of frozen *E. coli* stock were placed in a 50 mL falcon tube with 15 mL of RPMI Media 1640 (Gibco™, Invitrogen, Vienna, Austria) with 15% heat-inactivated fetal bovine serum (FBS) (Gibco™, Invitrogen, Vienna, Austria). *E. coli* DH5*α* cultures were grown at 37 °C under aerobic conditions for 24 h in an orbital shaker at 200 rpm, to ensure proper aeration. The bacteria were harvested by centrifugation at 10,000×g for 10 min, + 4 °C, collecting the cell pellet. Cells were re-suspended in 300 mL of 0.9% NaCl with protease inhibitors (Roche, Vienna, Austria) and divided into two aliquots. Cells from one aliquot were subjected to 3 rounds of sonication (Bandelin Sonopuls HD2070, Bandelin Electronic, Germany) with an amplitude of 50%, for 30 s with 30 s of cooling on ice in between rounds. The other aliquot was kept on ice without subjecting the cells to lysis. When complete, both cell aliquots were pooled together to achieve a mixture of proteins and intact bacteria in a single sample. This sample was then mixed vigorously with 1.2 mL of cold acetone (-20 °C) by vortexing and incubated on ice for 30 min. The cell-protein pellet was collected by centrifugation at 10,000×g for 10 min at + 4 °C. The pellet was once again re-suspended in acetone (-20 °C) and incubated on ice for 10 min. The cell-protein pellet was collected by centrifugation at 10,000×g for 10 min, + 4 °C, air-dried to remove the acetone, and pulverized with a sterile spatula to make a fine powder. The powder was stored at −20 °C until further use. For pre-absorption of sera, *E. coli* DH5*α* acetone powder was used in a final concentration of 1% (w/v) and incubated with sera overnight at + 4 °C with end-over-end rotation. After incubation, sera were centrifuged at 10,000×g for 10 min at + 4 °C, the supernatant was collected and used as the primary antibody for further protocols. The pre-absorption efficiency was examined with dot-blots, comparing pre-absorbed and non-pre-absorbed sera.

### Dot-blotting

The efficiency of the serum pre-absorption and the best serum concentration for Western Blotting and immunodetection were determined based on the dot-blot analysis. Furthermore, the dot-blots were used to confirm the presence of anti-*H. meleagridis* antibodies in the selected sera before applying them in the immunoprecipitation experiments. To prepare *E. coli*- protein for dot-blots, *E. coli* DH5*α* was cultivated in 15 mL of RPMI Media 1640 (Gibco™, Invitrogen, Vienna, Austria) with 15% FBS in 50 mL falcon tubes, as described above. After 24 h of growth, the cells were harvested by centrifugation at 10,000xg for 10 min at + 4 °C. The bacterial pellet was resuspended in Triton X-100 lysis buffer (50mM Tris/HCl, 150mM NaCl, 1mM EDTA, 1% Triton X-100) at pH 7.4, supplemented with Complete protease inhibitors cocktail (Roche Diagnostics GmbH, Vienna, Austria) and lysed by sonication (3 × 30 s cycles with continuous power). The supernatant containing soluble proteins was collected by centrifugation at 20,000×g for 10 min at + 4 °C. Protein concentration was measured using 2-D Quant Kit (Cytiva, Pasching, Austria) following the manufacturer’s instructions, and the aliquots were stored at -80 °C until further use.

To obtain *H. meleagridis*-protein for dot-blots, the protozoan culture was incubated, purified, and collected as described above. Cells were lysed in Triton X-100 lysis buffer supplemented with Complete protease inhibitors cocktail (Roche Diagnostics GmbH, Vienna, Austria) by using TissueLyser II (Qiagen, Hilden, Germany) for 2 rounds of homogenization (2 min at 28 Hz). The cell lysate was clarified by centrifugation at 10,000×g for 2 min at + 4 °C, and the supernatant containing soluble proteins was collected. Protein concentration was measured using 2-D Quant Kit (Cytiva, Pasching, Austria), and the aliquots were stored at -80 °C until further use.

Dot-blots were prepared on PVDF membranes (Pall Corporation, VWR International GmbH, Vienna, Austria) with 3 spots each for both the *H. meleagridis* and bacterial protein preparation using 100 µg, 10 µg and 1 µg of protein.

### Immunoprecipitation

For each sample,165µL (~ 5mg) of Dynabeads™ M280 Tosylactivated (Invitrogen, Thermo Fisher Scientific, Vienna, Austria) were used. Pre-absorbed sera from 3 different chickens or turkeys, vaccinated and infected with *H. meleagridis*, were diluted 1:1000 and then mixed in equal parts (1:1:1 ratio). One pool was made for immune sera originating from chickens and one for the turkey immune sera. Each aliquot of the beads was chemically conjugated with 300µL of the pooled sera through an overnight incubation at 37°C in 0.1 M borate buffer (pH9.5), with end-over-end rotation as suggested by the manufacturer. After conjugation, the beads were pulled down using a magnetic rack, and the supernatant was discarded. The beads were resuspended in 200µl of PBS buffer, pH7.4, containing 0.5% (w/v) BSA, and incubated for one hour at 37°C, with end-over-end rotation, to ensure their full coverage with ligands. Using once again the magnetic rack, the beads were isolated, and the supernatant was discarded. The beads were washed twice with 100µl of PBS solution, pH7.4, ensuring the removal of any unattached ligands. As a final step, the beads were re-suspended in 200µL of PBS buffer, pH7.4, and stored at +4°C (Fig. 7b).

**Fig. 8:**
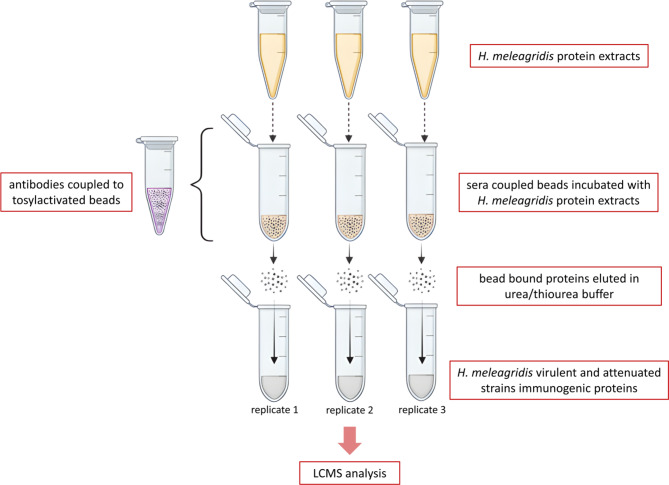
Schematic representation of the immunoprecipitation protocol for the isolation of immunogenic proteins of* H. meleagridis* . Beads with the attached antibodies were incubated with *H. meleagridis* protein extracts, allowing the immunogenic proteins to bind selectively. After the binding phase, the proteins attached to the beads were washed and eluted using a urea/thiourea buffer. The entire procedure is performed in triplicate to ensure reproducibility. Finally, eluates were analyzed using LCMS to identify the immunogenic proteins present in both the virulent and attenuated strains.

For immunoprecipitation, 500µL of the *H. meleagridis*-antigen preparation were added to the antibody-conjugated beads and incubated overnight at +4°C with end-over-end rotation. Following binding, beads were washed 3 times with 100µL PBS solution, pH7.4. The bead-bound protein complexes were eluted in 250µL elution buffer (7 M Urea/2 M Thiourea, 4%CHAPS, 1%DTT) by overnight incubation at +4°C with end-over-end rotation. The collected supernatant containing the *H. meleagridis* immunoproteome was analyzed on a silver-stained SDS-PAGE and Western Blot. Additionally, an aliquot was subjected to liquid chromatography-mass spectroscopy (LCMS) for further characterization (Fig. 8). To control for unspecific binding to the tosylactivated magnetic beads, two negative controls were included in the trials and processed. The first control consisted of the beads that were coated only with BSA. This ensured the full coverage of the beads with BSA and tested for cross-reactivity between *H. meleagridis* proteins and BSA. A second control was performed with uncoated beads. By skipping the ligand binding step, we tested whether unspecific binding of proteins to the beads would occur omitting the specific temperature and buffer conditions described above. Similar to samples from immunoprecipitation, samples from both negative controls were analyzed on a silver-stained SDS-PAGE and subjected to LCMS for protein identification.

To control for the sera cross-reactivity, the same protocol was carried out using sera from naïve chickens and turkeys without *H. meleagridis* infection. Briefly, sera from 3 different chickens or turkeys, never exposed to *H. meleagridis*, were pooled, and chemically conjugated to Dynabeads™ M280 Tosylactivated (Invitrogen, Thermo Fisher Scientific, Waltham, MA, USA). Further incubation with the same antigen preparations was performed as described above. Bound proteins were eluted in 250µL elution buffer (7 M Urea/2 M Thiourea, 4%CHAPS, 1%DTT), one aliquot was analyzed on silver-stained SDS-PAGE, and the other was subjected to LCMS for protein identification.

### Sodium dodecyl sulfate–polyacrylamide gel electrophoresis (SDS-PAGE) and western blotting

For each immunoproteomic (IP) preparation, 20µL of the sample was mixed with 5µL of SDS-PAGE loading buffer and subjected to electrophoresis on a 12% SDS-PAGE gel for 90 min at a constant 120V. Each IP sample was analyzed in two SDS-PAGE gels. In one gel, the separated proteins were visualized using the silver-staining protocol^49^. The second gel was used for Western Blotting. For that purpose, proteins were transferred to a polyvinyl difluoride (PVDF) membrane (Pall Corporation, VWR International GmbH, Vienna, Austria) using the Trans-Blot Turbo Transfer System (Bio-Rad Laboratories, Vienna, Austria). After blotting, the PVDF membranes were blocked overnight at +4°C in 5% skim milk dissolved in 0.05% Tween 20-containing PBS solution, pH7.4. Afterwards, the membranes were incubated for 1 h with the 1:1,000 dilution of pre-absorbed pool of immune sera from chicken or turkey on a rocker platform at room temperature. After washing the primary antibody, membranes were incubated with the secondary antibody, a horseradish conjugated donkey anti-chicken IgY (IgG) (1:20,000 dilution; Jackson ImmunoResearch Laboratories, Inc., Dianova, Hamburg, Germany) or goat anti-turkey IgG (1:20,000 dilution; Southern Biotech, Biomedica, Vienna, Austria) for one hour at room temperature. After washing off the secondary antibody, the immune reaction was detected with Pierce™ ECL Western Blotting Substrate (Pierce, Thermo Fisher Scientific, Vienna, Austria).

### Identification of immunogenic proteins by liquid chromatography-mass spectroscopy (LCMS)

Samples with immunogenic proteins of *H. meleagridis* virulent and attenuated strains obtained by immunoprecipitation with either chicken or turkey sera were subjected to LCMS for identification. The sample’s protein concentrations ranged between 0.5 and 0.7µg/µL. Tryptic digest was performed using a filter-aided sample preparation protocol according to^50^. After C18 cleanup, resulting peptides were analyzed on a nano-HPLC Ultimate 3000 RSLC system (Dionex) coupled to a high-resolution Q-Exactive HF Orbitrap mass spectrometer (Thermo Fisher Scientific)^50^. For quantitative analysis samples were injected in technical duplicates. Proteome Discoverer Software 2.4.1.15 (Thermo Fisher Scientific) was used for the identification of proteins based on the acquired peptide spectra. The protein databases used for the searches included the in-house annotated *H. meleagridis* proteome database^15^ as well as UP_Ecoli_tx83333.fasta, UP_turkey_tx9103.fasta or UP_chicken_tx9031.fasta, and crap.fasta (https://www.thegpm.org/crap/) (remaining UP downloaded from UniProt 09/22). For database searches following parameters were applied: enzyme trypsin, maximum of two missed cleavage sites, precursor mass tolerance 10 ppm and fragment mass tolerance 0.02 Da, dynamic modifications: oxidation (+15.995 Da on methionine), deamidation (+0.984 Da on asparagine and glutamine), Gln-> pyro-Glu (-17.027 Da on glutamine), and several N-terminal modifications (acetylation, met-loss, and met-loss + acetyl), static modification: carbamidomethyl (+57.021 Da on cysteine). Identified proteins were further filtered for proteins identified with at least two identified peptides and at least one unique, identified peptide.

Intensity-based label-free quantification was performed based on protein abundance values (normalized to total area sums) generated with the Proteome Discoverer software. Normalized abundance values of the technical replicates were aggregated by the mean before statistical analysis with R version 4.2.0 (R Core Team, 2022). Data quality was further improved by excluding proteins with one or two missing abundance values within the three biological replicates from further analyses. For testing for statistically significant abundance changes between the two groups, a two-sample t-test was used. Proteins with significant changes in abundance level reported as up- or downregulated were identified with more than two tryptic peptides and quantified with at least one unique peptide and a fold change higher/lower than ±2 fold with a *p*-value lower than 0.05 adjusted according to Benjamini-Hochberg for controlling the false discovery rate.

### In silico analysis

Immunogenic proteins identified by LCMS were analyzed to determine their putative antigenicity using the VaxElan software (https://vac.kamalrawal.in/vaxelan/v2, accessed on 12 January 2023) and their potential for being used as a vaccine candidate using the VaxiDL software (https://vac.kamalrawal.in/vaxidl/, accessed on 13 January 2023). For antigenicity prediction, the VaxElan software was set with the following parameters: adhesion property^51^, CTL epitope prediction^52^, gene essentiality^53^, molecular weight^54^, non-bacterial pathogenicity^55^ and virulence factors^56^. In the present study, a protein was considered antigenic when presenting a positive result for at least 50% of the tested features, giving a VaxElan score ≥ 0.5. VaxiDL. Software search to determine vaccine candidates was run with default settings and the organism model - protozoa.

## Electronic supplementary material

Below is the link to the electronic supplementary material.

Supplementary Material 1

Supplementary Material 2

## Data Availability

The mass spectrometry proteomics data have been deposited to the ProteomeXchange Consortium via the PRIDE^57^ partner repository with the dataset identifier: PXD041172. For data requests, please contact the corresponding author, Dr. Ivana Bilic (ivana.bilic@vetmeduni.ac.at).
